# Validation of *In utero* Tractography of Human Fetal Commissural and Internal Capsule Fibers with Histological Structure Tensor Analysis

**DOI:** 10.3389/fnana.2015.00164

**Published:** 2015-12-24

**Authors:** Christian Mitter, András Jakab, Peter C. Brugger, Gerda Ricken, Gerlinde M. Gruber, Dieter Bettelheim, Anke Scharrer, Georg Langs, Johannes A. Hainfellner, Daniela Prayer, Gregor Kasprian

**Affiliations:** ^1^Division of Neuroradiology and Musculoskeletal Radiology, Department of Biomedical Imaging and Image-guided Therapy, Medical University of ViennaVienna, Austria; ^2^Institute of Neurology, Medical University of ViennaVienna, Austria; ^3^Computational Imaging Research Lab, Department of Biomedical Imaging and Image-guided Therapy, Medical University of ViennaVienna, Austria; ^4^Department of Systematic Anatomy, Center for Anatomy and Cell Biology, Medical University of ViennaVienna, Austria; ^5^Division of Obstetrics and Feto-maternal Medicine, Department of Obstetrics and Gynecology, Medical University of ViennaVienna, Austria; ^6^Clinical Institute for Pathology, Medical University of ViennaVienna, Austria

**Keywords:** human fetal brain, diffusion tensor imaging, tractography, fetal MRI, structure tensor, histology, validation

## Abstract

Diffusion tensor imaging (DTI) and tractography offer the unique possibility to visualize the developing white matter macroanatomy of the human fetal brain *in vivo* and *in utero* and are currently under investigation for their potential use in the diagnosis of developmental pathologies of the human central nervous system. However, in order to establish *in utero* DTI as a clinical imaging tool, an independent comparison between macroscopic imaging and microscopic histology data in the same subject is needed. The present study aimed to cross-validate normal as well as abnormal *in utero* tractography results of commissural and internal capsule fibers in human fetal brains using postmortem histological structure tensor (ST) analysis. *In utero* tractography findings from two structurally unremarkable and five abnormal fetal brains were compared to the results of postmortem ST analysis applied to digitalized whole hemisphere sections of the same subjects. An approach to perform ST-based deterministic tractography in histological sections was implemented to overcome limitations in correlating *in utero* tractography to postmortem histology data. ST analysis and histology-based tractography of fetal brain sections enabled the direct assessment of the anisotropic organization and main fiber orientation of fetal telencephalic layers on a micro- and macroscopic scale, and validated *in utero* tractography results of corpus callosum and internal capsule fiber tracts. Cross-validation of abnormal *in utero* tractography results could be achieved in four subjects with agenesis of the corpus callosum (ACC) and in two cases with malformations of internal capsule fibers. In addition, potential limitations of current DTI-based *in utero* tractography could be demonstrated in several brain regions. Combining the three-dimensional nature of DTI-based *in utero* tractography with the microscopic resolution provided by histological ST analysis may ultimately facilitate a more complete morphologic characterization of axon guidance disorders at prenatal stages of human brain development.

## Introduction

The development of the human fetal brain involves a precisely orchestrated sequence of events, from proliferation of neuronal and glial precursor cells within the germinal zones, to migration toward their prospective targets in the cortical plate or deep nuclei, and finally the organization and wiring into both local as well as long-range neuronal networks (Sidman and Rakic, 1973; Marin and Rubenstein, 2003; Bystron et al., 2008; Judaš, 2011). As the molecular mechanisms of axon guidance and synaptogenesis are being gradually uncovered in animal models (Fame et al., 2011; Grant et al., 2012; Molnár et al., 2012), new developmental frameworks for human brain malformations will increasingly incorporate knowledge about axon guidance defects and pathological white matter connectivity into their classification schemes (Fallet-Bianco et al., 2008; Edwards et al., 2014).

Fetal MRI has established itself as an important adjunct to ultrasound in the prenatal assessment of both normal brain development as well as central nervous system pathologies (Girard et al., 1995; Prayer et al., 2006; Prayer, 2011). Advanced MRI techniques like diffusion tensor imaging (DTI) and tractography (Basser et al., 1994; Mori et al., 1999) offer the unique possibility to investigate the three-dimensional anatomy of white matter fiber tracts *in vivo*, and have successfully been used to visualize the corpus callosum, the internal capsule, and even association fibers in the developing fetal brain *in utero* during the second and third trimester (Kasprian et al., 2008; Mitter et al., 2011, 2015). The ability to non-invasively assess abnormal connectivity in brain malformations, as has been shown for Probst bundles in cases of agenesis of the corpus callosum (ACC; Kasprian et al., 2013; Jakab et al., 2015a), demonstrates the potential of this novel technique for the prenatal diagnosis of white matter fiber tract pathologies and disorders of axon guidance. However, a more regular inclusion of DTI in clinical fetal MR protocols (Mailath-Pokorny et al., 2012; Jakab et al., 2015b) also underlines the need for an independent cross-validation of *in utero* DTI and tractography results, as well as insights about their limitations.

Until recently, validation of DTI-based tractography results in the adult human brain relied primarily on gross dissection studies using the Klingler fiber dissection technique (Ludwig and Klingler, 1956; Fernández-Miranda et al., 2008a,b; Martino et al., 2011), or direct comparison to myelin-stained histological sections (Bürgel et al., 2006). Newer histological techniques such as 3D-polarized light imaging (Axer et al., 2011a,b,c) or serial optical coherence scanner imaging (Wang et al., 2014a,b) are able to map the three-dimensional course of axons in postmortem human brain tissue based on the birefringence of myelin sheaths. Unfortunately, all of these techniques require the presence of myelin and are therefore not suited for validation of tractography results in the unmyelinated human fetal brain. Three-dimensional microscopy techniques that rely on rendering tissue transparent (Chung et al., 2013) are to date limited to small tissue volumes, and thus might be impractical for the systematic analysis of whole hemispheres in the human fetal brain. In animal models, DTI results can be microscopically validated by direct autoradiographic axonal tract tracing (Schmahmann and Pandya, 2006; Schmahmann et al., 2007). However, axonal tracing in the human fetal brain, using lipophilic dyes like DiI, is time-consuming, limited to relatively short distances and challenging, since the dye has to be manually placed within a region of interest (Hevner, 2000).

To date, histological correlates of *in utero* (Kasprian et al., 2013) and postmortem DTI results in the human fetal brain have been identified by visual comparisons of imaging results to histological sections (Ren et al., 2006; Saksena et al., 2008; Trivedi et al., 2009a,b; Vasung et al., 2010, 2011; Widjaja et al., 2010; Huang et al., 2013; Xu et al., 2014). Due to its much higher resolution, postmortem DTI (Huang et al., 2006, 2009; Takahashi et al., 2012; Kolasinski et al., 2013) serves as an important anatomical reference for *in utero* DTI studies, but does not in itself solve the problem of validation.

Structure tensor (ST) analysis is an image processing approach that enables the directional analysis of fibers in histological sections on a microscopic scale (Rezakhaniha et al., 2012), and has already been used to study the microstructural white matter anatomy in whole-hemisphere sections of the adult human brain (Budde and Annese, 2013). In ST analysis, a ST is computed from a local neighborhood to derive the local orientation, anisotropy, and intensity for every pixel of an image. The results have been shown to correlate well to analogous quantitative measures of fiber orientation and fractional anisotropy derived from DTI (Budde and Frank, 2012). Importantly, ST analysis does not require the presence of myelin and can be performed on immunohistochemically stained sections, making it feasible for histology-based validation of DTI in the human fetal brain.

Due to multiple sources of artifacts and the limited resolution of *in utero* DTI, the corresponding tissue structures of *in utero* tractography findings, as well as ultimately their clinical relevance, are currently unclear. In order to establish and strengthen DTI as a diagnostic and prognostic prenatal imaging tool, we aimed at investigating the postmortem histological ST correlates of both normal and abnormal *in utero* tractography results for commissural and internal capsule fibers in seven fetal subjects. To further optimize the postmortem validation of DTI-based tractography (Budde et al., 2011), and to allow for a better macroscopic correlation, we implemented an approach to perform deterministic tractography on digitalized histological sections based on data derived from ST analysis. In addition, we utilized ST analysis to demonstrate the anisotropic organization and main fiber orientation of the transient fetal layers that can be identified in the telencephalic wall during the second trimester (Kostovic et al., 2002; Bystron et al., 2008) on a microscopic scale.

## Material and methods

### Subjects

Histopathological specimens from seven human fetal brains were included in this study, in which 1.5 or 3 Tesla *in utero* DTI had been performed within a time span of 2 weeks prior to fetal demise (mean = 10 days). Imaging was performed during clinically indicated fetal MR examinations to confirm sonographically detected fetal or extrafetal pathologies. In each case, parental counseling regarding termination of pregnancy followed a multidisciplinary conference (radiologists, obstetricians, neuropediatricians). Postmortem human brain tissue was obtained during routine neuropathological autopsies of the fetal brain after termination of pregnancy for medical reasons. Fetal subjects were 21–28 gestational weeks of age (GW, calculated from the first day of the woman's last menstrual cycle and determined with reference to a previous sonography examination) at the time of fetal MRI and 22–29 GW at the time of death. In two cases (subjects 1 and 6) neuropathological autopsy revealed normal brain development with no signs of cerebral malformations. The remaining five were fetuses with malformations of the developing white matter, including four cases of ACC and one case of Joubert syndrome. For a more detailed description of fetal pathologies see Table 1. All imaged mothers gave written, informed consent for the fetal MRI examination and the study was approved by the Ethics Committee of the Medical University of Vienna (EK Nr.:650/2010).

**Table 1:** **Neuropathology and extracranial pathologies of investigated fetal subjects**.

**Subject #**	**Gestational age at time of fetal MRI (in gestational weeks + days)**	**Gestational age at time of death**	**Neuropathology**	**Extracranial pathologies**
Subject 1	20 + 2	21 + 3	No signs of cerebral malformations	Placental hypoplasia, small for gestational age, coarctation of the aorta
Subject 2	20 + 1	22 + 0	Joubert syndrome with hypoplastic vermis and molar-tooth configuration of the mesencephalon, heterotopic projection of the pyramidal tract into the interpeduncular cistern with interpeduncular white matter heterotopia and absence of the pyramidal tract caudal to the mesencephalon, normal anatomy of corpus callosum and internal capsule	Multicystic kidneys
Subject 3	20 + 4	22 + 1	Complete ACC, abnormal gyri and sulci in both frontal lobes, periventricular and subcortical gray matter heterotopia, malrotation of the right hippocampus, disorganized fiber architecture in the left corona radiata	No extracranial malformations
Subject 4	22 + 0	22 + 4	Complete ACC, subependymal heterotopia, hippocampal malrotation, cerebellar hypoplasia	Hypertelorism
Subject 5	22 + 0	23 + 5	Complete ACC, abnormal gyri and sulci in both frontal lobes, malformation of the left frontal lobe with disturbed lamination of the telencephalic wall, abnormal white matter architecture, and enlarged ganglionic eminence, midline cyst	Hypertelorism, epicanthic folds
Subject 6	25 + 1	26 + 3	No signs of cerebral malformations	Congenital muscular dystrophy, pulmonary hypoplasia, cardiac dilatation
Subject 7	27 + 1	29 + 0	Complete ACC, abnormal gyri and sulci in the right frontal lobe, delayed sulcation in the temporal lobes	Parvovirus infection, no extracranial malformations

### Fetal MR imaging protocol and *In utero* tractography

**Figure 1:**
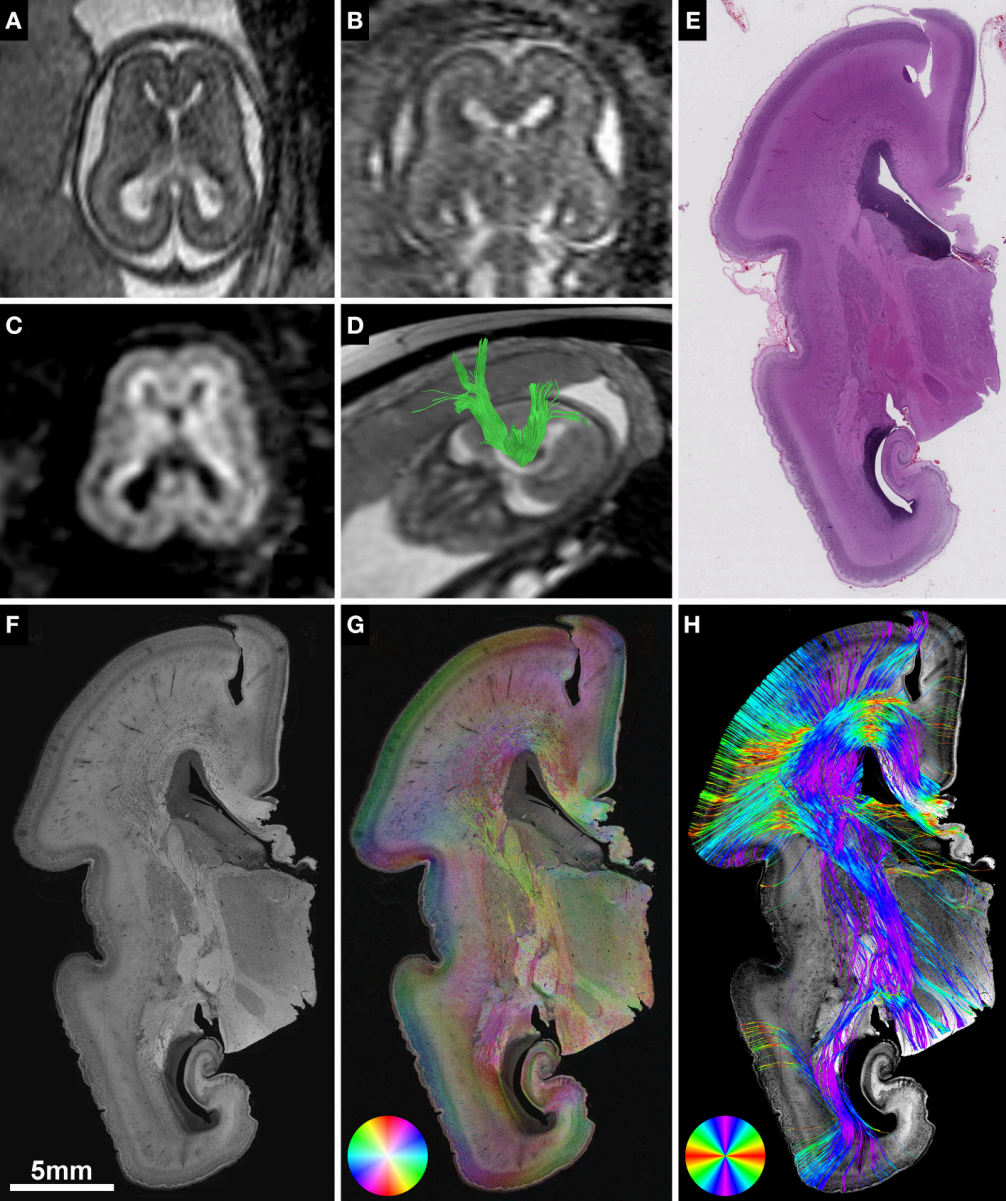
**Validation of *in utero* tractography with structure tensor analysis of histological sections**. **(A–D)** Fetal MRI of subject 2 at GW21. **(A)** Axial and **(B)** coronal T2-weighted images. *In utero* DTI **(C)** based tractography **(D)** visualizes the internal capsule (green) in both hemispheres. **(E–H)** Coronal histological sections of the right hemisphere at the level of the posterior limb of the internal capsule in the same subject at GW22. **(E)** H&E staining for anatomical reference. **(F)** Inverted grayscale immunohistochemistry for NCAM to identify axonal tracts. **(G)** Fiber orientation, anisotropy and staining intensity derived from ST analysis are displayed in a color-coded hue-saturation-brightness (HSB) image, respectively. **(H)** Histology-based tractography uses this information to reconstruct streamlines for better macroscopic comparison with the results of *in utero* tractography, with hue representing fiber orientation. Please note the different color-coding schemes for HSB images and histology-based tractography.

Fetal imaging was performed in six cases on a 1.5 T MR system (Philips Gyroscan) using a five-channel phased-array cardiac coil, adjusted to the position of the fetal head. There was no sedation used. An echo planar diffusion tensor sequence was acquired in an axial plane perpendicular to the axis of the brainstem using 16 gradient-encoding directions, *b*-values of 0 and 700 s/mm^2^ and a reconstructed asymmetric voxel size of 0.94/0.94/3 mm. One case (subject 5) underwent diagnostic 3 T MRI and was retrospectively included (reconstructed voxel size 2.01/2.01/4 mm, *b*-values of 0 and 700 s/mm^2^, six gradient-encoding directions). Axial T2-weighted sequences were acquired as anatomical references for tractography (Figures 1A–C).

*In utero* tractography was performed using Philips Extended MR WorkSpace 2.6.3.3 according to the protocols described previously (Kasprian et al., 2008, 2013; Mitter et al., 2015). Briefly summarized, a multiple region of interest approach was used with a minimum of two regions of interest placed within the projection path of the fiber tract of interest. Fiber tracts were visualized using a deterministic linear tracking algorithm with an FA threshold of 0.15 and a maximum angle change of 27.0° (Figure 1D). The fiber tracts visualized with *in utero* tractography included the internal capsule in all subjects, the corpus callosum in the three subjects with normal commissural anatomy, and the Probst bundles in the four subjects with ACC. See Kasprian et al. (2008) and Kasprian et al. (2013) for details and images of region of interest placement.

### Neuropathological procedures and immunohistochemistry

Histological specimens used for this study were obtained from the brain bank of the Institute of Neurology, Medical University of Vienna, Austria. In all cases a routine neurofetopathological autopsy had been performed after termination of pregnancy for medical reasons. Fetal brains were fixed in 10% buffered formaldehyde solution (0.9% sodium chloride, 0.3% zinc sulfate heptahydrate) for at least 2 weeks, cut in approximately 5 mm thick coronal slices (brainstem and cerebellum were cut in the axial plane) and embedded in paraffin. Tissue blocks at the level of the basal ganglia and thalamus were cut at 3–5 μm and automated immunohistochemistry was performed on Autostainer 48 Link instruments (Dako, Glostrup, Denmark), using EnVision™ FLEX+ detection system (#K8002 Dako, Glostrup, Denmark) according to manufacturer's recommendations. Briefly, sections were deparaffinized in xylene and heat-induced epitope retrieval was performed in EnVision™ FLEX Target Retrieval Solution, low pH (#K8005 Dako, Glostrup, Denmark) at 95°C for 20 min. Axonal tracts were identified with antibody against neural cell adhesion molecule (NCAM) (Jakovcevski et al., 2007). Primary antibody NCAM (#MON9006-1 Monosan, Uden, The Netherlands) was used at a dilution of 1:200 and incubated for 30 min at room temperature. After automated immunohistochemistry, sections were dehydrated through graded alcohols and mounted. Adjacent sections were stained with hematoxylin and eosin (H&E) for anatomical reference (Figure 1E).

### Digitalization and structure tensor analysis of histological sections

NCAM-stained sections were digitalized using an automated slide scanner (Hamamatsu NanoZoomer 2.0-HT) at 40X magnification with a scanning resolution of 0.23 μm. For further image processing, digital images were down-sampled, depending on the size of the region of interest, to magnification levels of 20X, 10X, or 5X, converted into 8-bit grayscale and inverted (Figure 1F). ST analysis (Budde and Frank, 2012; Rezakhaniha et al., 2012) was performed with the *OrientationJ* plugin for ImageJ (Sage, 2012) using a cubic spline gradient with a Gaussian window of 5 pix. The results were visualized as hue-saturation-brightness (HSB) images, with hue representing the orientation, saturation the anisotropy/coherency, and brightness the staining intensity (Figure 1G).

### Histology-based tractography in digitalized sections

Digitalized histological data with a 10X magnification were transferred to the image-processing workstation in JPEG format. Prior to tractography analysis, pre-processing was carried out using customized software in the Matlab environment (MATLAB and Statistics Toolbox Release R2010a, The MathWorks, Inc., Natick, Massachusetts, United States.). The red channel of the red-green-blue color-coded digital images was converted into 8-bit grayscale values. In order to optimize the image contrast, histogram equalization was performed (Contrast-limited adaptive histogram equalization using the *adapthisteq* command in Matlab R2010a), and values were normalized to a range of 0-256. ST analysis (Budde and Frank, 2012) was estimated using the *Toolbox Diffc* (Peyre, 2008) in Matlab R2010a. The output of the estimated ST was saved as three scalar images in NIFTI image format. The calculation speed was enhanced by using parallelized block-processing program architecture in Matlab, which utilized eight CPU cores.

In addition to the ST, the two-dimensional fractional anisotropy (FA_2*d*_) and eigenvalues (λ_1_ and λ_2_) were also calculated and saved in NIFTI format. The calculation of the FA_2*d*_ utilized the following formula:

$$FA_{2d}=\frac{\sqrt{{\left(\lambda_{1}-\bar{\lambda }\right)}^{2}+{\left(\lambda_{2}-\bar{\lambda }\right)}^{2}}}{\sqrt{{\left(\lambda_{1}+\lambda_{2}\right)}^{2}}}$$

where λ_1_ and λ_2_ are the eigenvalues of the structure tensor D, and $\bar{\lambda }=\frac{\lambda_{1}+\lambda_{2}}{2}$.

The eigenvalues and FA_2*d*_ maps were used to generate a mask of the brain in the cross-sections, and a seed mask for initiating tractography. We separated the background and foreground using an in-house-developed code Matlab R2010a, and the resulting image mask was used to restrict the FA_2*d*_ image to the areas depicting the brain only. Within the brain mask, the top 95th percentile of FA_2*d*_ values was calculated and a high-pass thresholding was performed.

The two-dimensional STs were given by three scalar images, which were then generalized to three dimensions by replacing the Z components with zeros:

$$D_{pseudo}=\left[\begin{array}{cc} G_{XX} & G_{XY}\\ G_{XY} & G_{YY} \end{array}\right]=\left[\begin{array}{ccc} G_{XX} & G_{XY} & 0\\ G_{XY} & G_{YY} & 0\\ 0 & 0 & 0 \end{array}\right]$$

where *D*_pseudo_ is the pseudo-diffusion tensor, derived from the 2D STs, and G_XX_, G_XY_, and G_YY_ are components of the 2D ST.

The outputs of the ST, FA_2*d*_, and the mask calculation step were down-sampled by a factor of 4 to enable feasible utilization of classical fiber tractography algorithms. The diffusion tensor was saved as a 4D-NIFTI file containing six scalar images, determined from the upper triangle of the matrix form of *D*_*pseudo*_, and these data were directly accessible by image processing toolboxes for the analysis of diffusion tensor images. Histology-based tractography was performed by the Diffusion Toolbox in the CAMINO software environment (Cook et al., 2006).

Deterministic histology-based tractography was initiated from the masks covering the top FA_2*d*_ values of the white matter, and tractography was terminated if streamlines reached out of the estimated 2D brain mask. A fourth-order Runge-Kutta fiber-tracking method (rk4) was utilized with probabilistic nearest neighbor interpolation of streamlines, similar to the interpolation algorithm described in Behrens et al. (2003), while the rk4 tracking approach is detailed in Basser et al. (2000) and further technical documentation is found at: http://cmic.cs.ucl.ac.uk/camino/. For each seed voxel, 10 iterations were performed, in steps of 2 pixels, and a curve threshold of 75° while tracking was also constrained to FA_2*d*_ values higher than 0.1. Streamlines were colored according to the principal eigenvector orientation, and were saved in VTK format for visualization (Figure 1H).

## Results

### ST analysis of fetal telencephalic layers in brains with normal anatomy of the internal capsule and corpus callosum

**Figure 2:**
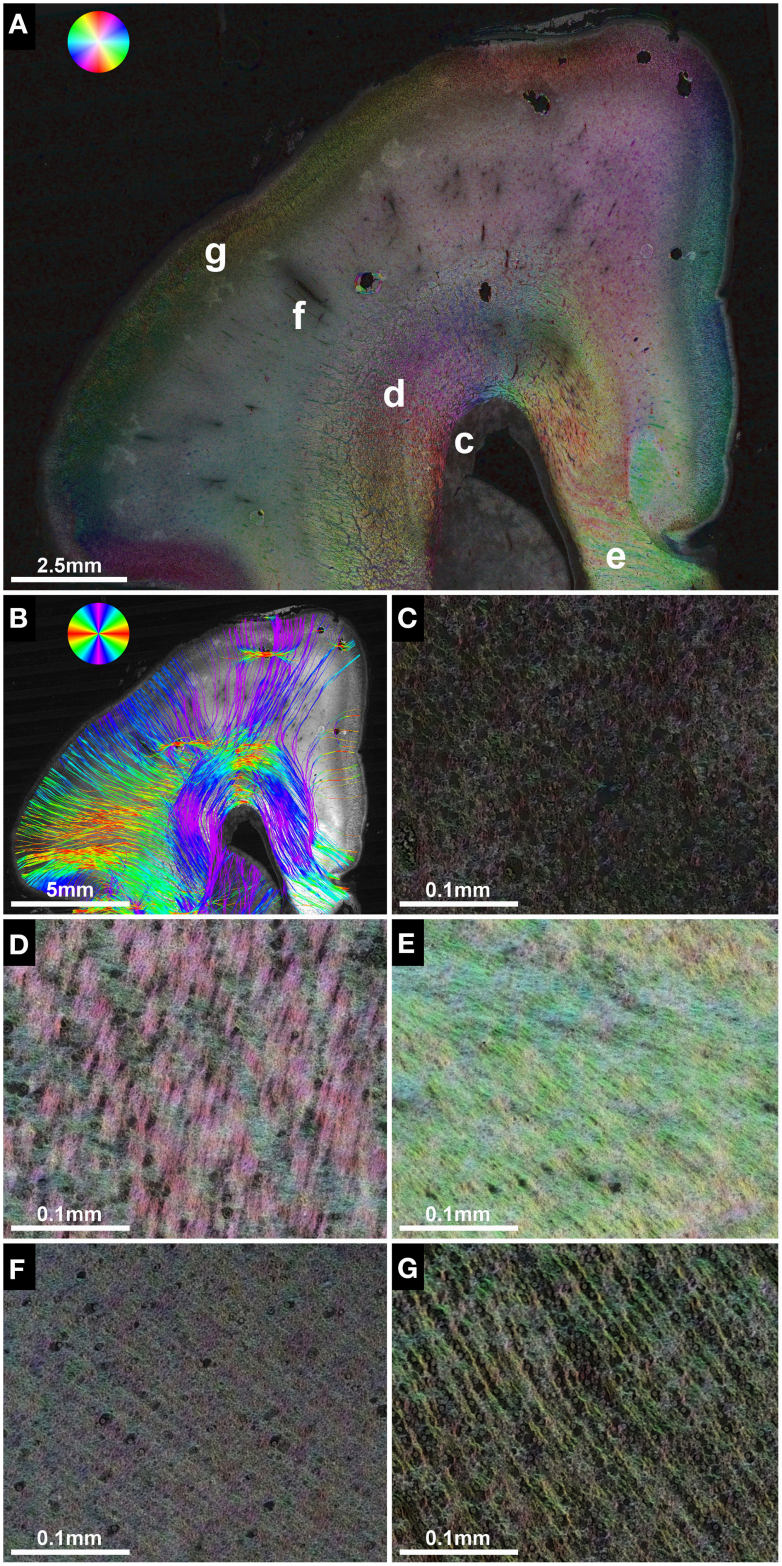
**Anisotropic organization and main fiber orientation of transient fetal layers in the telencephalic wall**. **(A)** HSB image and **(B)** histology-based tractography of the right parietal lobe in subject 1 at GW22, with no signs of cerebral malformations. Lower-case letters mark regions shown at higher magnification. **(C)** No apparent directional organization of the ventricular zone. **(D)** The intermediate zone demonstrates high anisotropy with a predominant tangential projection pattern of small discrete bundles of axons on a more diffusely organized background of radially oriented crossing fibers. **(E)** High anisotropy and tangential orientation of fibers in the corpus callosum. **(F)** Low anisotropy within the subplate. **(G)** High radial anisotropy in the cortical plate. Please note that the black holes in the cortical plate and subplate in **(A)** are artifacts due to damage during histological processing and may produce artificial results in histology-based tractography **(B)**.

**Figure 3:**
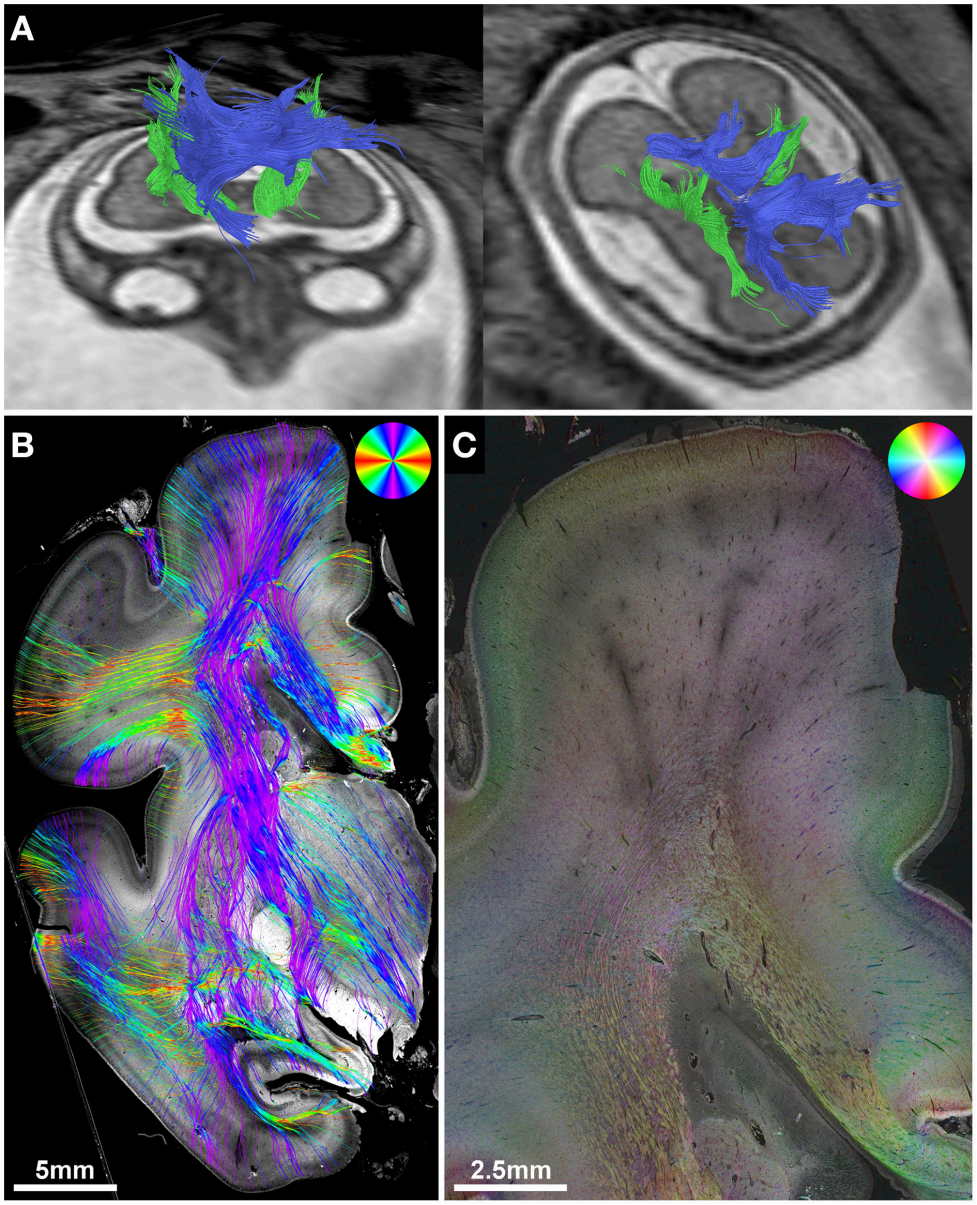
**Validation of *in utero* tractography of the internal capsule and corpus callosum**. **(A)** *In utero* tractography of the internal capsule (green) and corpus callosum (blue) in subject 6 at GW26. **(B)** Histology-based tractography on coronal sections through the PLIC of the right hemisphere at GW27 demonstrates the main fiber orientation of the corticospinal tract and corpus callosum. **(C)** Corresponding HSB image.

HSB images of the three fetal brains with normal anatomy of the internal capsule and corpus callosum (subjects 1, 2, and 6) demonstrated the anisotropic organization of the transient fetal layers of the telencephalic wall on a histological scale (Figures 1–**3**). Located immediately adjacent to the lateral ventricles, the ventricular zone showed a lower degree of immunoreactivity for NCAM compared to the other layers of the fetal telencephalon, with no apparent directional organization on HSB images (Figure 2C).

In contrast, the fetal intermediate zone contained abundant NCAM-positive fibers with a predominant tangential projection pattern of small discrete bundles of axons running parallel to the ventricular wall, and a more diffusely organized fraction of radially oriented crossing fibers (Figure 2D). The amount of fiber crossings within the intermediate zone changed gradually from lateral (corona radiata) to medial (corpus callosum), with a progressive decrease of radial fibers and a corresponding increase of tangentially oriented fibers (Figure 2E). Visualization of the intermediate zone with histology-based tractography displayed a dominant tangential fiber population, showing thick bundles of streamlines arching in a C-shape from the exit of the internal capsule around the lateral ventricle toward the corpus callosum (Figure 2B).

The subplate zone, located between the intermediate zone and the cortical plate, was characterized by low anisotropy with a diffuse network of crossing fibers and a weak radial organization seen at lower magnifications (Figure 2A). At higher magnifications, however, this was less evident (Figure 2F). Finally, the cortical plate showed a relatively strong anisotropy, with an arrangement of fibers radial to the cortical surface (Figure 2G). The radial organization of the cortical plate and subplate was represented on histology-based tractography images as streamlines projecting in a radial fashion from the intermediate zone toward the surface of the brain (Figure 2B).

### *In utero* identification of the internal capsule, corpus callosum, and probst bundles with DTI-based tractography

**Figure 4:**
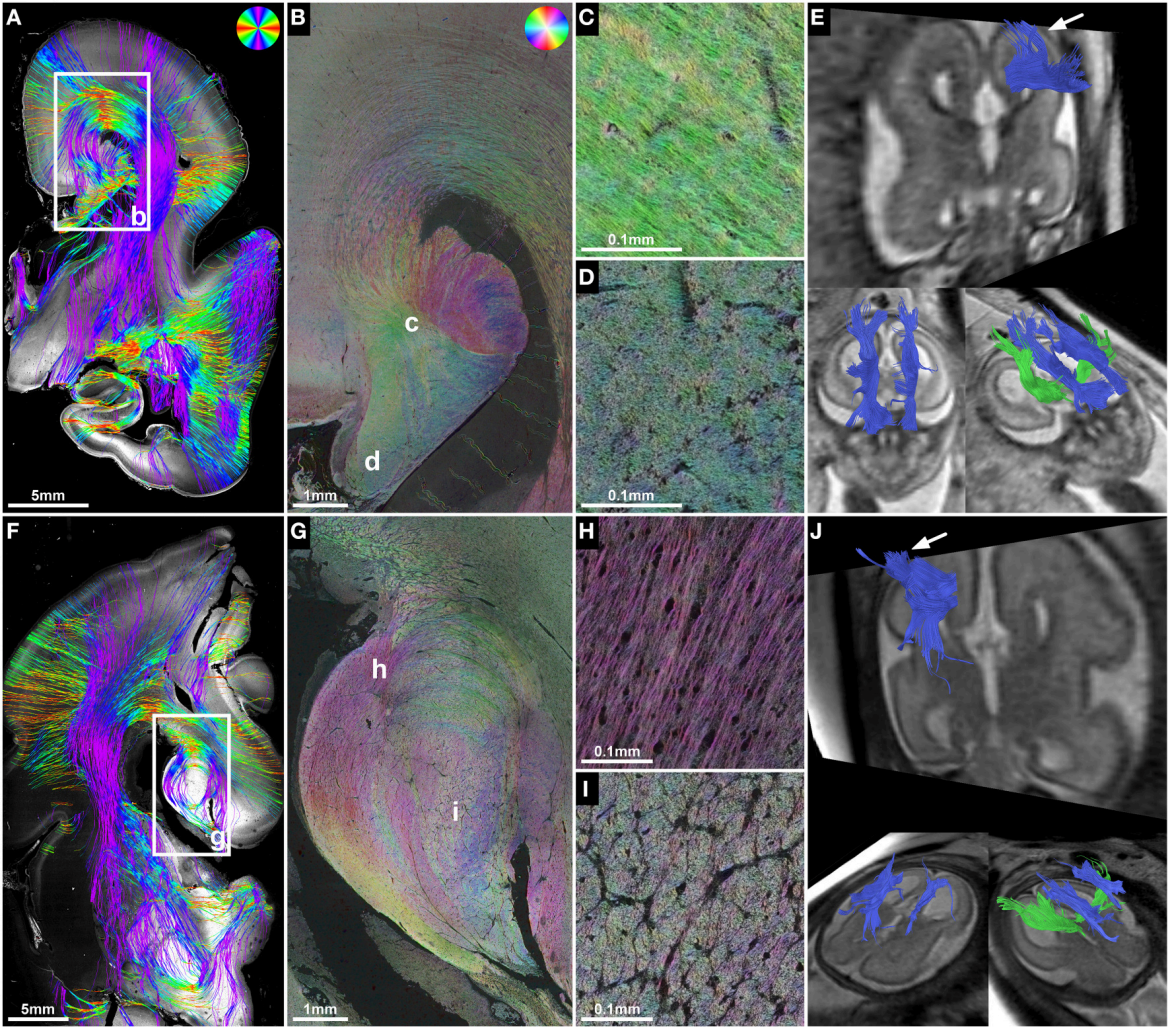
**ST analysis reveals the internal fiber architecture of Probst bundles in subjects with ACC**. Histology-based tractography of subject 4 at GW23 **(A)** and subject 7 at GW29 **(F)** depicts streamlines projecting from the intermediate zone into the Probst bundle. **(B,G)** ST analysis of Probst bundles reveals their internal structure as a complex arrangement of both longitudinal **(C,H)** and anterior-posteriorly oriented perpendicularly **(D,I)** cut fibers. **(E,J)** Corresponding to the histological ST findings, *in utero* tractography of subject 4 at GW22 and subject 7 at GW28 visualized not only anterior-posteriorly oriented fibers within the Probst bundles (blue), but also bundles of streamlines along its course that diverged superiorly into the telencephalic wall (arrow).

**Figure 5:**
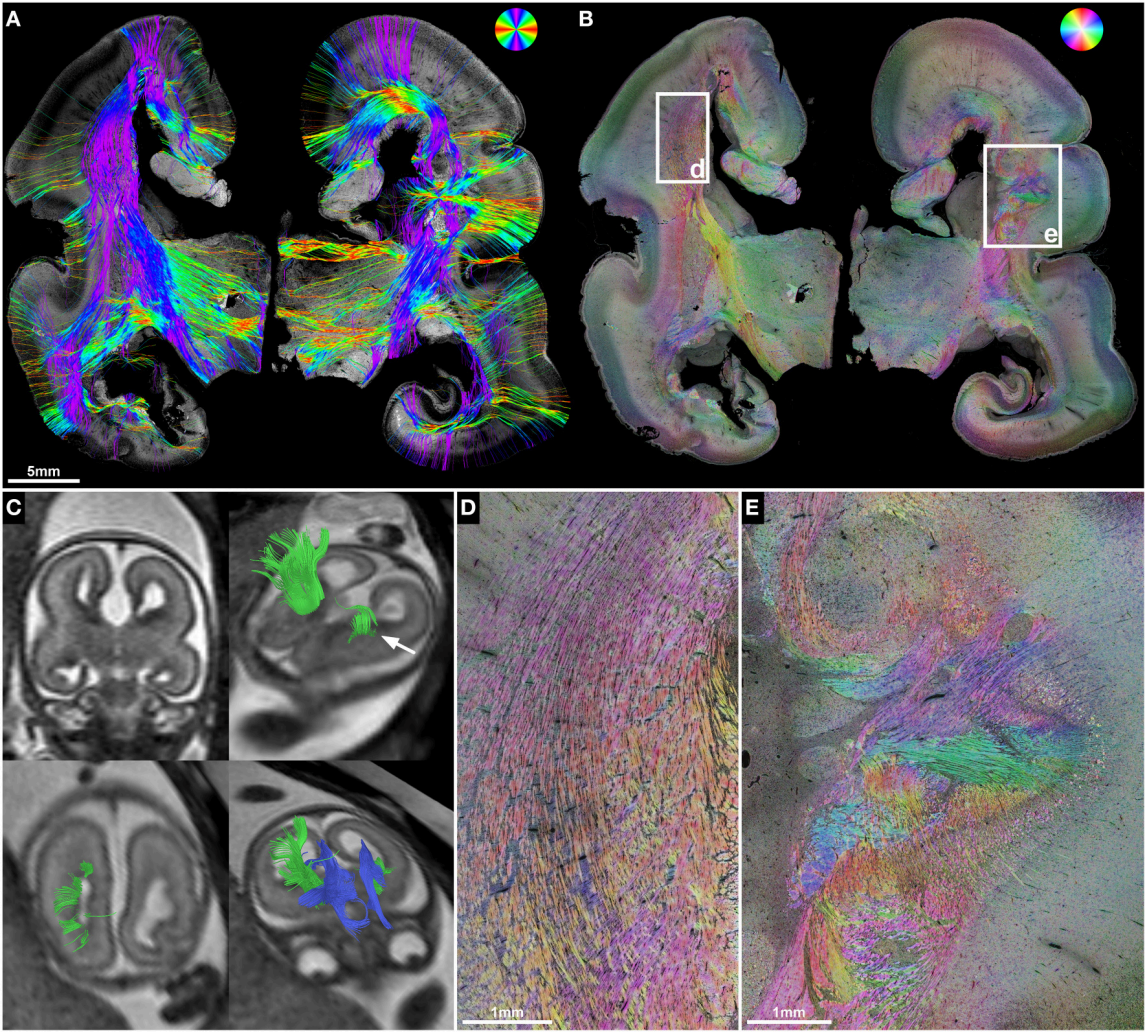
**ST validation of abnormal *in utero* tractography results of the internal capsule**. **(A)** Histology-based tractography and **(B)** HSB image of coronal sections through the PLIC in subject 3 at GW23 demonstrate complete ACC and orientation of main fiber tracts. **(C)** *In utero* tractography at GW21 visualizes bilateral Probst bundles (blue) and a pronounced asymmetry of internal capsule fibers (green), with normal results for the right hemisphere and a discontinuity of streamlines in the left hemisphere after exiting the PLIC (arrow). HSB images at higher magnification validate the *in utero* findings by visualizing a regular corona radiata anatomy in the right hemisphere **(D)**, and a convolution of abnormally oriented fibers in the left hemisphere, with interspersed gray-matter heterotopia **(E)**.

*In utero* tractography of the corpus callosum was successful in three subjects with a mediolateral tangential projection pattern of fibers in the roof of the lateral ventricles. Further lateral, corpus callosum streamlines were traced that diverged radially from the intermediate zone in a U-shape toward the cortical plate (Figure 3A). In the four subjects with ACC, no mediolateral streamlines could be visualized. Instead, massive anterior-posteriorly oriented abnormal fiber tracts, corresponding to the Probst bundles, were found medial to the lateral ventricle in both hemispheres (Figures 4E,J). *In utero* tractography of the internal capsule was successful in all seven subjects in both hemispheres, with fibers showing a predominant inferior-superior projection within the posterior limb of the internal capsule (PLIC) between the basal ganglia and the thalamus. Further superior, internal capsule streamlines projected in a straight inferior-superior direction through the corona radiata toward the superolateral convexity, with a general stop of streamlines above the level of the corpus callosum or Probst bundles (Figure 3A). In subject 3, tractography results were asymmetric, with a discontinuity of left internal capsule streamlines at the exit of the PLIC into the corona radiata (Figure 5C). Results are summarized in Table 2.

**Table 2:** **Detection of internal capsule, corpus callosum and Probst bundles with DTI-based *in utero* tractography and histology-based tractography**.

**Subject #**	**DTI-based *in utero* tractography**		**Histology-based tractography**	
	**Internal capsule (IC)**	**Commissures**	**Internal capsule (IC)**	**Commissures**
Subject 1	Bilateral IC	Corpus callosum	Bilateral IC	Corpus callosum
Subject 2	Bilateral IC	Corpus callosum	Bilateral IC	Corpus callosum
Subject 3	Bilateral IC^*^	Bilateral Probst bundles	Bilateral IC	Commissural agenesis^***^
Subject 4	Bilateral IC	Bilateral Probst bundles	Bilateral IC	Commissural agenesis^***^
Subject 5	Bilateral IC	Bilateral Probst bundles	Bilateral IC	Commissural agenesis^***^
Subject 6	Bilateral IC	Corpus callosum	Right IC^**^	Corpus callosum in right hemisphere^**^
Subject 7	Bilateral IC	Bilateral Probst bundles	Bilateral IC	Commissural agenesis^***^

*For detailed description see text*.

* *Discontinuity of streamlines in the left hemisphere at the transition of the PLIC into the corona radiata*.

** *In subject 6 histology was only available for the right hemisphere*.

*** *Absence of normal mediolaterally oriented corpus callosum projections, successful tractography of longitudinally cut fibers, and reduced number of streamlines in regions of the Probst bundle with predominantly perpendicularly cut axons*.

### Validation of *In utero* tractography of the internal capsule and corpus callosum with histology-based tractography

Corresponding to the *in utero* tractography results for the internal capsule, histology-based tractography of NCAM-stained sections successfully visualized tangentially cut fibers of the internal capsule and corona radiata in all subjects (Table 2). Streamlines were located between the basal ganglia and thalamus and projected in an inferior-superior direction through the PLIC and intermediate zone lateral to the lateral ventricle. While some of the fibers diverged radially toward the cortical plate, most of the intermediate zone streamlines followed the lateral ventricular wall in a tangential fashion (Figures 1H, 3B).

Although the corpus callosum was artificially torn during autopsy in subjects 1, 2, and 6, histology-based tractography confirmed the *in utero* tractography finding of normal interhemispheric connections with the visualization of thick bundles of mediolaterally oriented fibers along the roof of the lateral ventricle. Streamlines continued laterally into the tangentially oriented fibers of the intermediate zone, with some fibers diverging radially through the subplate toward the cortical plate (Figures 1H, 2B, 3B). In the younger fetal brains (subjects 1 and 2 at GW22) intermediate zone streamlines projected in a C-shape around the lateral ventricle (Figures 1H, 2B). In contrast, in subject 6 at GW27 the directional change of intermediate zone streamlines around the lateral ventricle followed a more acute angle (Figure 3B).

### ST analysis of probst bundles and validation of *In utero* tractography results in cases of ACC

ST analysis of coronal histological sections successfully identified bilateral perpendicularly cut Probst bundles in all four subjects with ACC, thereby confirming the results of *in utero* tractography (Table 2). In addition, ST analysis was able to visualize the internal fiber architecture of Probst bundles, revealing a complex arrangement of both perpendicularly, and longitudinally cut fibers (Figure 4). While the center of the Probst bundle consisted of large amounts of perpendicularly cut fibers with high NCAM staining intensity, but low anisotropy within the coronal plane (Figures 4D,I), the lateral and dorsal portions contained a large number of longitudinally cut fibers, especially at the border to the intermediate zone, with a relatively high anisotropy (Figures 4C,H).

Although histology-based tractography cannot directly visualize off-plane fibers, an indirect correlate of anterior-posteriorly projecting Probst bundle fibers was found as a reduction of streamlines in areas with predominantly perpendicularly cut fibers (Figure 4F). In addition, histology-based tractography visualized the longitudinally cut fiber component as streamlines projecting from the intermediate zone into the Probst bundle (Figures 4A,F). Histological tractography results of fibers projecting in or out of the Probst bundles corresponded to *in utero* tractography results, which showed bundles of streamlines diverging from an anterior-posterior course within the Probst bundle superiorly into the telencephalic wall (Figures 4E,J).

### ST analysis in malformations of internal capsule fibers

The potential of histological ST analysis for the postmortem validation of malformations of internal capsule fibers, including the corona radiata and corticospinal tract, could be demonstrated in several subjects.

*In utero* tractography of subject 3 (GW21, ACC with bilateral Probst bundles) revealed a normal-appearing right-hemispheric internal capsule that could be traced through the intermediate zone into the central region of the developing cortex. In the left hemisphere however, internal capsule streamlines stopped at the transition of the PLIC into the corona radiata (Figure 5C). Neuropathological examination at GW23, with histological ST analysis of coronal sections at the level of the PLIC, demonstrated a pronounced malformation of the left corona radiata, with convoluted and abnormally oriented bundles of fibers, and interspersed gray-matter heterotopia within the intermediate zone (Figures 5B,E). In contrast, coronal sections of the right hemisphere at the level of the PLIC showed a normal pattern of predominantly tangentially oriented corona radiata fibers lateral to the right lateral ventricle (Figures 5B,D). Histology-based tractography was able to validate the *in utero* DTI-based tractography results, with streamlines from the right PLIC extending in a straight inferior-superior direction through the intermediate zone into the cortical plate, while the left corona radiata showed a chaotic arrangement of inferior-superiorly and mediolaterally oriented streamlines (Figure 5A).

**Figure 6:**
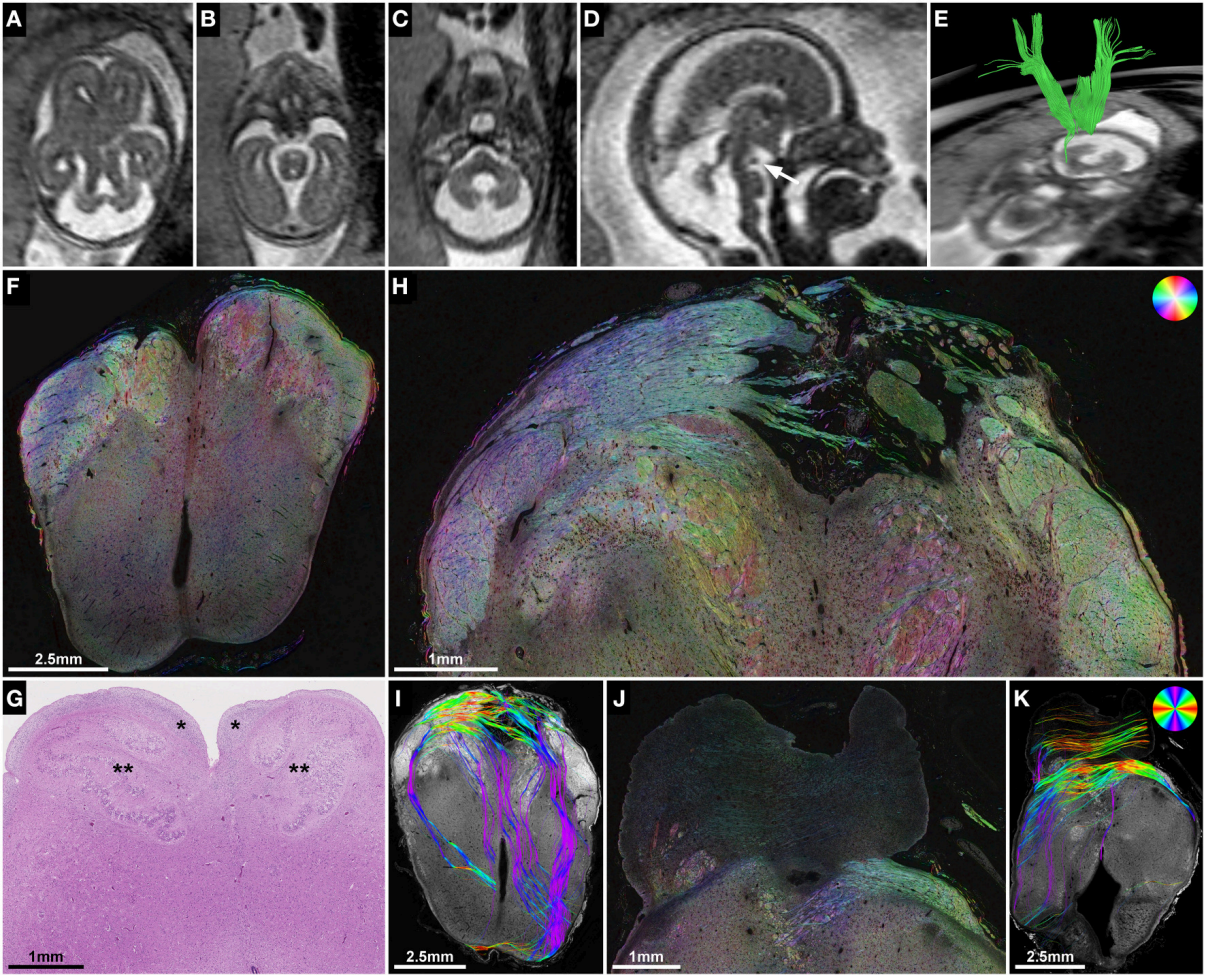
**ST analysis of heterotopic corticospinal tract projections in Joubert syndrome**. **(A–E)** Fetal MRI of subject 2 at GW21. Axial T2-weighted images through the brainstem demonstrate molar tooth malformation of the mesencephalon **(A)** and vermian hypoplasia **(C)**. **(D)** A midsagittal T2-weighted image shows a nodular hypointense interpeduncular mass (arrow). **(E)** *In utero* tractography shows a discontinuity of most internal capsule streamlines at the level of the mesencephalon. **(F)** Neuropathological autopsy at GW22 demonstrated perpendicularly cut fibers in the cerebral peduncles at the level of the rostral mesencephalon, but showed a complete absence of the corticospinal tract in the medulla oblongata **(G)** between the arcuate nuclei (^*^) and the inferior olive (^**^). **(H,I)** Sections through the caudal mesencephalon show a heterotopic projection of the corticospinal tract from the cerebral peduncles medially into the interpeduncular cistern. **(J,K)** Sections through the pons visualize some transverse pontine fibers and abnormal fiber tracts in the pontine tegmentum and posterior right basis pontis. Note the absence of normal perpendicularly cut fibers of the corticospinal tract between the pontine nuclei. **(F,H,J)** HSB images, **(I,K)** Histology-based tractography, **(G)** H&E.

Subject 2 demonstrated the potential use of ST analysis in fetal brains with malformations of descending corticospinal tract projection fibers. In this case fetal MRI at GW21 revealed typical features of Joubert syndrome, including molar tooth malformation of the mesencephalon (Figure 6A), vermian hypoplasia (Figures 6C,D) and multicystic kidneys (not shown). *In utero* tractography showed a normal appearance of the internal capsule within the cerebral hemispheres, but a stop of most streamlines at the level of the cerebral peduncles (Figure 6E). Neuropathological examination at GW22 confirmed the unremarkable anatomy of the internal capsule on coronal H&E-sections, HSB images, and histology-based tractography of the cerebral hemispheres (see Figure 1), but revealed pronounced abnormalities of fiber tracts within the brainstem. While the cerebral peduncles could be readily identified at the level of the rostral mesencephalon (Figure 6F), axial sections caudal to the mesencephalon showed an absence of normal descending fiber tracts, with no identifiable corticospinal tract between the pontine nuclei (Figure 6J) and a complete absence of the pyramids at the level of the medulla oblongata (Figure 6G). ST analysis of axial sections at the level of the caudal mesencephalon revealed an abnormal projection of thick bundles of longitudinally cut axons from the cerebral peduncles ventromedially, where fibers broke through the surface of the brain and formed an irregular formation of heterotopic white matter fiber bundles within the interpeduncular cistern (Figure 6H). Histology-based tractography provided similar results, with multiple mediolaterally oriented streamlines projecting from the cerebral peduncles into the interpeduncular subarachnoid space (Figure 6I). Additional neuropathological abnormalities in this brain included an asymmetric configuration of the basis pontis with a ventral pontine cleft, bilateral fragmented dentate nuclei (not shown) and abnormal heterotopic fiber bundles within the pontine tegmentum and posterior right basis pontis (Figures 6J,K). Finally, retrospective analysis of T2-weighted fetal MR images revealed an interpeduncular mass in the location of the histologically demonstrated white matter heterotopia (Figures 6B,D).

**Figure 7:**
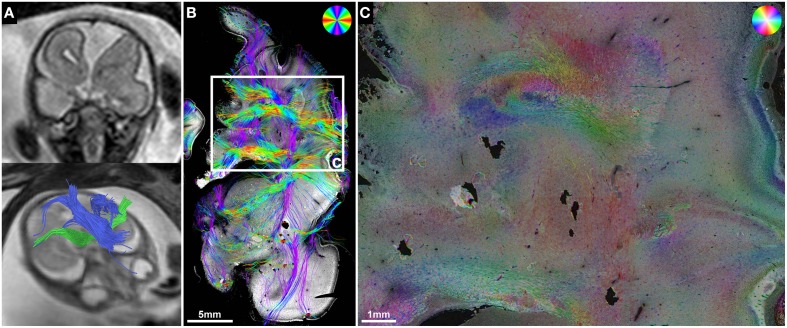
**ST analysis reveals limitations of current *in utero* DTI**. **(A)** Fetal MRI of subject 5 at GW22 shows a malformation of the left frontal lobe, complete ACC with Probst bundles and unremarkable internal capsules. **(B)** Histology-based tractography and **(C)** HSB image of the left frontal lobe at GW24 visualized a convolution of abnormally oriented fiber bundles high above the left lateral ventricle frontal horn that was not represented by the *in utero* tractography results.

In subject 5, neuropathological autopsy confirmed the fetal MRI findings of complete ACC with bilateral Probst bundles and left frontal lobe malformation with abnormal sulcation (Figure 7A). In addition, HSB images visualized a convolution of abnormally oriented fiber bundles high above the left lateral ventricle frontal horn (Figure 7C), which resembled intermediate-zone fibers at higher magnification, and were visualized by histology-based tractography as a chaotic arrangement of predominantly mediolaterally oriented streamlines (Figure 7B). *In utero* tractography in this case resulted in unremarkable visualization of the internal capsule in both hemispheres with a stop of streamlines at the level of the Probst bundles and no abnormal fiber tracts detected in the left frontal lobe (Figure 7A).

## Discussion

This retrospective study demonstrates the validity of *in utero* tractography results of commissural and internal capsule fibers in the developing human fetal brain by correlating DTI findings with the results of postmortem histological ST analysis (Budde and Frank, 2012; Budde and Annese, 2013) in the same subjects. ST analysis successfully validated abnormal *in utero* tractography findings of commissural fibers in subjects with ACC, and revealed additional information about the internal fiber architecture of Probst bundles. The potential for cross-validation of abnormal *in utero* tractography results of internal capsule fibers was demonstrated in subjects with malformation of the corona radiata and heterotopic projection of the corticospinal tract. Potential limitations of DTI-based *in utero* tractography could be demonstrated in several brain regions. While *in utero* DTI can currently achieve in-plane resolutions down to 1 mm (Kasprian et al., 2008, 2013; Mitter et al., 2015; Jakab et al., 2015a), and postmortem fetal DTI is usually performed with a resolution of several hundred μm (Huang et al., 2009; Vasung et al., 2011; Takahashi et al., 2012, 2014; Kolasinski et al., 2013), the measurement of anisotropic water diffusion within an imaged voxel provides only an indirect estimation of macroscopic fiber orientation. In contrast, ST analysis of histological fetal brain sections enables the direct assessment of the anisotropic organization of the developing fetal brain on a microscopic scale.

### Histological ST analysis enables the direct assessment of the anisotropic organization and fiber orientation of fetal telencephalic layers on a micro- and macroscopic scale

The main fiber orientation and anisotropy of the transient fetal telencephalic layers (Kostovic et al., 2002; Bystron et al., 2008), as demonstrated by HSB images, corresponds well to the results of DTI in postmortem fetal brains, which depict the cortical plate and subplate as layers of high and low radial anisotropy, respectively, and the intermediate zone as a layer of high tangential anisotropy (Huang et al., 2006). ST analysis results for the corona radiata are particularly interesting, as histological studies have shown that the fetal intermediate zone at the exit of the internal capsule between the caudate nucleus and the putamen contains a high amount of crossing fibers, including thalamocortical, corticofugal, commissural, and external capsule fibers (Judaš et al., 2005; Vasung et al., 2010). HSB images at high magnification clearly visualize these complex “periventricular crossroads” (Kostovic and Judas, 2006; Kostovic et al., 2014) of discrete tangential axon bundles and more diffusely organized radial crossing fibers. Since DTI cannot resolve crossing fibers within a single voxel, and instead depicts the average main diffusion direction, the inferior-superiorly oriented streamlines visualized by *in utero* tractography within the corona radiata represent the dominant tangential projection pattern of thalamocortical and corticofugal axons (Kostovic and Goldman-Rakic, 1983; Kostovic and Rakic, 1984; Ulfig et al., 2000). Likewise, histology-based tractography emphasizes the dominant tangential fiber components throughout the entire intermediate zone of the central region. Superior to the lateral angle of the lateral ventricle, *in utero* tractography generally failed to visualize tangential fibers and instead emphasized internal capsule and corpus callosum fibers that diverge radially toward the cortical plate, resulting in a straight inferior-superior shape of internal capsule streamlines and a U-shape of the corpus callosum. This resulted most likely from the acute angle change of tangential intermediate zone fibers in this region, especially in older fetal brains.

### Histological ST analysis allows the validation of abnormal *In utero* tractography results of internal capsule fibers

The case of interpeduncular heterotopia demonstrates the complementary nature of *in utero* tractography and postmortem histological ST analysis. An interpeduncular mass in patients with Joubert syndrome was first described in a neuroradiological case series (Harting et al., 2011), but due to a lack of neuropathological correlation the exact nature of this heterotopic tissue remained unclear. In subject 2, neuropathological analysis revealed the interpeduncular mass seen on fetal MRI to be composed of the abnormal heterotopic projection of the descending corticospinal tract into the interpeduncular cistern, a finding well visualized by HSB images and histology-based tractography. Of note, the neuropathological phenotype in Joubert syndrome also contains features of axon guidance disorders (Engle, 2010) with a non-decussation of the superior cerebellar peduncles and corticospinal tracts seen in many subjects (Friede and Boltshauser, 1978; Yachnis and Rorke, 1999; Ferland et al., 2004; Juric-Sekhar et al., 2012). Although the results of ST analysis in this case correspond well to the *in utero* tractography findings of a discontinuity of internal capsule streamlines at the level of the cerebral peduncles, it must be noted that *in utero* tractography of fiber tracts within the fetal brainstem remains a challenge, even in the normal developing brain (Kasprian et al., 2010).

Periventricular nodular and subcortical heterotopia fall into the spectrum of neuronal migration disorders (Barkovich et al., 2012) and are associated with several of the many known ACC syndromes (Edwards et al., 2014). HSB images and histology-based tractography in subject 3 clearly demonstrated the effect of gray matter heterotopia and chaotic intermediate zone fiber orientation on the ability to trace normal inferior-superiorly oriented corona radiata streamlines on histological sections. ST analysis thus validates the *in utero* tractography findings of a discontinuity of streamlines in the left hemisphere and an unremarkable appearance of the internal capsule in the right hemisphere.

### Histological ST analysis can be used to visualize the internal fiber architecture of probst bundles in cases of ACC

Although histology-based tractography cannot directly visualize anterior-posteriorly oriented Probst bundle fibers, the absence of normal mediolateral corpus callosum projections in all subjects with ACC provides indirect proof of the *in utero* tractography findings. In addition, high-resolution HSB images revealed the complex internal structure of Probst bundles with centrally arranged perpendicularly cut fibers and longitudinally cut fibers in the lateral and dorsal portions of the bundle. This is in line with the original description of the “Balkenlängsbündel” by Moriz Probst (Probst, 1901). *In utero* tractography streamlines that diverge from their main anterior-posterior projection pattern into a superior direction toward the telencephalic wall may represent the imaging correlate of these hemispheric projections into the Probst bundles. This interpretation is further supported by corresponding results of histology-based tractography.

### Insights into possible limitations of *In utero* DTI

In addition to valuable insights about the diagnostic possibilities of current fetal DTI sequences, ST analysis may also reveal important clues about their limitations. In subject 5, HSB images and histology-based tractography were able to demonstrate a complex convolution of abnormally oriented fiber bundles within the left frontal lobe that *in utero* tractography failed to visualize. Potential contributing factors that might explain the inability of *in utero* tractography to trace these fibers include resolution limitations as well as marked angle changes within convoluted fiber tracts. The well-known inability of DTI to resolve crossing fibers (Mori and Tournier, 2013) may contribute to the failure of *in utero* tractography to visualize tangential intermediate zone fibers superior to the lateral angle of the lateral ventricle, as mentioned above. Furthermore, it prohibits the direct evaluation of radial crossing fibers within “periventricular crossroad” regions of the corona radiata (Judaš et al., 2005).

### Limitations of histological ST analysis

Histological ST analysis has important limitations that need to be considered when correlating its findings to the results of DTI-based tractography. First, while DTI acquires a volume dataset and can therefore be used to trace fibers in three dimensions, ST analysis is limited to the two-dimensional images provided by light microscopy. The resulting drawback is an inability to directly characterize perpendicularly cut fibers (Budde and Frank, 2012; Budde and Annese, 2013) and affects in coronal sections predominantly anterior-posteriorly oriented fiber tracts such as the center of the Probst bundles, the fornix, the cingulum, the optic radiation as well as most long association fiber tracts within the lateral hemisphere. However, limitations concerning perpendicularly cut fibers in the coronal plane could be overcome by using either axially or sagittally cut sections. Although it is possible to extend ST analysis to the third dimension by acquiring serial image stacks with confocal microscopy (Khan et al., 2015), such approaches are to date limited to small tissue volumes and thus might be impractical for the systematic analysis of whole-hemisphere human fetal brain sections.

Because of the two-dimensional nature of ST analysis, it is important to emphasize that histology-based tractography is not equivalent to direct axon tracing, since even longitudinally cut fibers usually travel only short distances within a 4 μm thick histological section. Depicted streamlines therefore represent compound results of multiple similarly oriented fiber bundles and, like the results of DTI-based tractography, should be rather viewed as visualizations of macroscopic white matter anatomy. In our study population fetal MRI was performed within a time span of 2 weeks prior to death, which means that developmental changes during that time must be taken into account when correlating histology to MR images.

Finally it should be noted that postmortem damage to the brain during autopsy or histological processing may produce artificial results in histology-based tractography with either abnormally projecting streamlines or a stop of fibers. Histological sections should therefore be carefully screened for signs of artificial damage prior to interpretation of tractography results.

## Conclusion

Despite these difficulties, histology-based tractography of commissural and internal capsule fibers in NCAM-stained human fetal brain sections corresponds remarkably well to many of the results of *in utero* tractography. As *in utero* tractography has now been extended from the corpus callosum, the internal capsule (Kasprian et al., 2008) and Probst bundles (Kasprian et al., 2013; Jakab et al., 2015b) to even cortico-cortical association fiber tracts (Mitter et al., 2011, 2015), histological ST analysis may serve as a valuable tool in the postmortem validation and neuropathological examination of a wide range of both normal and pathological axon fiber tracts in the developing human brain. Aside from the retrospective validation of *in utero* tractography in case of fetal demise, a combined approach that complements the three-dimensional nature of DTI with the microscopic resolution provided by histological ST analysis may improve our understanding of abnormal white matter neuroanatomy in disorders of axon guidance at prenatal stages of human brain development.

## Author contributions

CM, AJ, and GK designed the research; CM, PCB, GR, GMG, DB, AS, JAH, DP, and GK collected the data; CM, AJ, and GL analyzed the results; CM, AJ, PCB, GR, and GK wrote the manuscript text. All authors reviewed the manuscript.

### Conflict of interest statement

The authors declare that the research was conducted in the absence of any commercial or financial relationships that could be construed as a potential conflict of interest.
